# The role of drug use in brief alcohol interventions: a multi-ethnic/racial analysis

**DOI:** 10.1186/1940-0640-7-S1-A18

**Published:** 2012-10-09

**Authors:** Craig Field, Gerald Cochran, Raul Caetano

**Affiliations:** 1 Health Behavior Research and Training Institute, University of Texas at Austin, Austin, TX, USA; 2 Center for Social Work Research, The University of Texas at Austin School of Social Work, Austin, TX, USA; 3 School of Health Professions, University of Texas Southwestern Medical Center, Dallas, TX, USA

## 

To advance the understanding of the role other drug use plays in alcohol brief intervention (BI), we examined the effects of baseline drug dependence on alcohol use outcomes and the effects of alcohol BI on drug use among injured patients. Hierarchical linear modeling was used to conduct a secondary analysis of data from a randomized trial of patients admitted to a Level-1 trauma center who screened positive for alcohol misuse. A series of two-level models were developed to test the interaction of drug dependence and treatment on alcohol use outcomes for Hispanic (n = 539), white (n = 667), and black (n = 287) patients and the effects of alcohol BI on drug use at 12 months. Results showed significant effects of BI on alcohol outcomes among Hispanic patients but not among white or black patients for percent days abstinent (six months: B = 0.27, SE = 0.10, p = 0.006; 12 months: B = 0.41, SE = 0.11, p < 0.001), volume per week (six months: B = -1.91, SE = 0.77, p = 0.01; 12 month: B = -2.71, SE = 0.86, p = 0.002), and maximum amount consumed per drinking occasion (six months: B = -1.08, SE = 0.46, p = 0.02; 12 months: B = -1.62, SE = 0.52, p = 0.002). Analysis for drug use as an outcome at 12 months showed no significant effects for any race/ethnicity group. In contrast to white and black patients, Hispanic patients with drug dependence who received alcohol BI were more likely to reduce drinking than those who received standard care. Alcohol BI did not appear to influence drug use at follow-up in any group. These results suggest drug use at baseline does not negatively influence drinking outcomes, and alcohol BI does not appear to influence drug use. Interventions specifically targeting drug use may be more likely to influence drug use.

